# Treatments for Primary Immune Thrombocytopenia: A Review

**DOI:** 10.7759/cureus.5849

**Published:** 2019-10-06

**Authors:** Margot Samson, William Fraser, David Lebowitz

**Affiliations:** 1 Emergency Medicine, University of Central Florida College of Medicine, Orlando, USA

**Keywords:** itp, hematology, literature reviews, immune thrombocytopenic purpura (itp)

## Abstract

Immune thrombocytopenic purpura (ITP) is an autoimmune condition that affects nearly 1:10,000 people in the world. It is traditionally defined by a platelet count of less than 100 x 10^9^L, but treatment typically depends on symptomology rather than on the platelet count itself. For primary idiopathic ITP, corticosteroids have been the standard first-line of treatment for symptomatic patients, with the addition of intravenous immune globulin (IVIG) or Rh_o_(D) immune globulin (anti-RhD) for steroid-resistant cases. In cases of refractory or non-responsive ITP, second-line therapy includes splenectomy or rituximab, a monoclonal antibody against the CD20 antigen (anti-CD20). In patients who continue to have severe thrombocytopenia and symptomatic bleeding despite first- and second-line treatments, the diagnosis of “chronic refractory ITP” is appropriate, and third-line treatments are evaluated. This manuscript describes the efficacy of different treatment options for primary ITP and introduces the reader to various third-line options that are emerging as a means of treating chronic refractory ITP.

## Introduction and background

Idiopathic thrombocytopenia or immune thrombocytopenia (ITP) is a hematological condition which is characterized by a low platelet count of less than 100 x 10^9^L. This platelet deficit can be caused by decreased production, immune-mediated destruction, or increased splenic sequestration of platelets, but typically involves autoantibodies to glycoproteins expressed on megakaryocytes, the precursor cell to platelets [1]. Symptoms of ITP can vary but tend to be symptoms of thrombocytopenia in general, such as petechiae, purpura, mucosal bleeding such as epistaxis, and in the most severe cases, fatal intracranial hemorrhage [2].

ITP is idiopathic in 80% of cases, and primary ITP is often thought of as an autoimmune condition [3]. However, 20% of cases of ITP can present secondary to coexisting illnesses [2]. For example, ITP is often seen after infection. In children, who account for half of the cases of ITP seen per year, two-thirds of cases are preceded by a febrile infectious illness [3,4]. Specific associations between ITP and Helicobacter pylori, cytomegalovirus, varicella-zoster virus, hepatitis C virus, and human immunodeficiency virus have been documented. ITP has also been linked to chronic lymphocytic leukemia (CLL) (1-5% of CLL patients), as well as many other autoimmune and rheumatologic conditions [3].

The epidemiology of ITP is diverse and heterogeneous. Primary ITP has a prevalence of 9.5:100,000 in adults with an incidence of 3.3:100,000 per year [2]. While the clinical presentation can vary, the predominant symptom is bleeding, and the severity of presentation can range from asymptomatic to intractable bleeding. The presentation can be acute, lasting less than three months, persistent, between 3-12 months, or chronic, lasting greater than 12 months [3].

The treatment guidelines described below are typically reserved for primary ITP, as childhood ITP tends to resolve on its own, and secondary ITP management is based on the underlying disorder [4]. However, in severe and refractory cases of secondary ITP, some of the guidelines for primary ITP can be used to stabilize the patient, while treatment for the underlying disorder is initiated [5].

Treatment is typically reserved for those with symptomatic ITP. The goal is to achieve a hemostatic platelet count, which is around 20-30 x 10^9^L, although this varies by person. According to the 1996 American Society of Hematology (ASH) evidence-based practice guidelines for treating ITP, treatment should be administered for any newly diagnosed patients when platelets are less than 30 x 10^9^L. The 2011 guidelines suggest that this objective cutoff is still a useful value, but the decision to treat should ultimately be determined by patient preference, severity of symptoms, and risk factors for bleeding [6].

## Review

First-line treatments

In adults, the primary treatment for ITP is corticosteroids. Dexamethasone and prednisone have been shown to modulate B-cell and dendritic cell activation, leading to a decrease in immune-mediated destruction of platelets [2]. Up to 80% of patients respond to steroids, though many of those people relapse after steroids are tapered. Prednisone, typically 1 mg/kg/d for two to four weeks, has long been the mainstay of therapy, but several recent studies have shown that high-dose dexamethasone is even more effective. A study in Hong Kong of 125 patients with initial platelet counts of less than 20 x 10^9^/L demonstrated that a single short course of dexamethasone, 40mg per day for four days, led to a stable platelet count greater than 50 × 10^9^/L in 50% of responders, and remained stable six months later [7]. Additionally, several studies in Italy found that four-six cycles of dexamethasone given at two-week intervals showed a response rate of 80-90% at 15 months [8]. A retrospective study of 100 patients found that the response rate for high-dose dexamethasone was significantly higher than for prednisone at 42.7% vs. 28.4%, respectively [9]. A prospective trial of 26 patients demonstrated similar results, where initial response rates (platelet count > 50 x 10^9^ per liter) between prednisone and dexamethasone were both 100%, but long-term remission was significantly more frequent with pulsed dexamethasone at 77% vs. 22% with daily prednisone [10].

Corticosteroids are considered safe for pregnant patients with ITP who need treatment as well [6]. It is clear that corticosteroids, and more specifically, high-dose dexamethasone, are an effective initial treatment for ITP. The side effect profile of corticosteroids, including weight gain, hypertension, and diabetes, can be an issue for some patients, but corticosteroids are still a generally safe and easily administered treatment, making them an appropriate first-line choice [11].

In steroid-resistant patients, the addition of intravenous immunoglobulin (IVIG) or Rhₒ(D) immune globulin (anti-RhD) can be used to enhance the treatment effect. In addition, these two treatments can be used in patients when corticosteroids are contraindicated [6]. IVIG is also indicated when platelet counts need to be raised rapidly, such as in cases of active and severe bleeding, and can be used in conjunction with corticosteroids in select patients. The typical dosing is 1 g/kg/day infusion for one-two days, though regimens can vary based on physician's preference [12]. A study of 19 patients with chronic ITP showed a response rate of 75% with IVIG, with a cessation of active bleeding if IVIG was administered within 12 hours of active bleeding and an increase in platelet count within one hour in 53% of patients [13]. A review of 28 studies showed that 64% of patients obtained a peak platelet count >100,000/mm^3, ^and 83% of patients obtained a peak platelet count >50,000/mm^3^ after IVIG [14]. However, sustained remission is uncommon. In addition, IVIG is expensive and can carry side effects such as anaphylaxis and renal and pulmonary insufficiency [4]. Anti-RhD can be useful in conjunction with corticosteroids in patients who are RhD positive. One study reports an efficacity of 50-70% in patients, while others report 37% efficacy [11,12]. However, side effects such as severe hemolysis, nausea, fever, headache have been reported in some patients, so care should be taken [11,12].

Second-line treatments

If a patient fails initial therapy and does not achieve complete remission, which happens in up to 70-90% of patients, splenectomy, or removal of the spleen to decrease splenic sequestration of platelets, has traditionally been the second-line treatment [15]. In fact, the ASH 2011 guidelines still recommend splenectomy as the next choice in therapy after failure of remission with corticosteroids, IVIG, and anti-RhD [6]. Because the dysfunctional platelets are destroyed prematurely by the spleen in patients with ITP, a splenectomy can theoretically prevent that destruction. Some studies show a 65-70% complete response (defined as the absence of significant bleeding) with a 60-70% long-term response [12,16,17]. One retrospective study found that 60% of patients had sustained remission after splenectomy [11]. Another retrospective study of 174 patients showed that 88% of patients had a good response after therapy, but 20% of those patients relapsed. The study also found that younger and corticosteroid-dependent patients tended to have a greater chance of obtaining a response after splenectomy [18]. Splenectomy is generally deferred for as long as possible in children with ITP, as most cases resolve spontaneously, and because infection with encapsulated bacteria post-splenectomy tends to be severe. However, surgery can still be performed in severe and symptomatic cases that last longer than one year. Response rates for children are similar to adults, with complete remission in 70-80% of cases [4]. Splenectomy can be performed open or laparoscopically. Response rates between the two are similar. Laparoscopic surgery has a longer operative time but tends to have shorter hospital stays, less post-operative pain, and a shorter postoperative recovery, and so it is generally recommended [19]. Splenectomy has been found to be safe even in patients with very low platelet counts, and prophylactic platelet infusion is generally not indicated [20]. Risks of splenectomy include risks of surgery, infection, bleeding, thrombosis, and relapse [12].

The monoclonal antibody against the CD20 antigen (anti-CD20), rituximab, is one new option for the treatment of chronic and persistent ITP. The standard dosing for rituximab in treating ITP is 375 mg/m^2^/week intravenously (IV) for four weeks [5]. Multiple studies have compared the effectiveness of splenectomy and rituximab in treating ITP. One retrospective study of 105 patients with primary ITP found that the splenectomy group had better outcomes compared to the rituximab group, with complete response rates at 82.8% versus 39.5% at three months and 81.0% versus 35.9% at 12 months respectively [21]. A quasi-experimental study of 143 patients determined that there was no significant difference in response rate between those who underwent splenectomy versus rituximab treatment [22]. A retrospective study in the United States of 222 patients found that patients treated with splenectomy had greater five-year freedom from relapse than patients treated with rituximab at 53.7% versus 14.96%, respectively [15]. One review showed that 50% of patients had an initial response to rituximab, which lasted at least six months in 30% of those patients and was maintained after five years in 20% [2]. Another review discussed combining rituximab with high-dose dexamethasone as an alternative first-line treatment for ITP and saw that remission rates were higher with the combination at 63% versus 35% with monotherapy at six months, and 53% versus 33% at one year. However, this increase in remission was accompanied by a higher incidence of severe toxicity. This study also described the efficacy of rituximab alone and reported initial response rates of 40-60% with a sustained response rate of 20% at five years [5]. Toxicity of rituximab in the short-term includes infusion reactions, serum sickness, and cardiac arrhythmias [12]. A review of several studies demonstrated that 21.6% of patients had a mild or moderate adverse event, typically infusion reactions, while 3.7% reported severe events, and 2.9% died. This suggests that rituximab is a potentially dangerous treatment and should be prescribed with caution [23]. In addition, these studies show that while rituximab is a promising new treatment, splenectomy is still more effective in patients who can tolerate the surgery. Therefore, rituximab remains an option for patients where splenectomy is contraindicated, as well as in children with persistent and severe ITP [6].

Third-line treatments

Patients who fail first-line therapy and still have no response after splenectomy have what is termed “chronic refractory ITP” [4]. These patients are only treated if they are at risk of severe bleeding. Many of these patients are re-treated with prednisone, though long-term use of corticosteroids is intolerable due to the many side effects discussed above [4]. The introduction of many new drugs has expanded the choice of treatment for chronic refractory ITP in recent years. In addition, these drugs may be an alternative in patients where splenectomy is contraindicated or has a higher risk, such as in children and pregnant patients. Studies are still ongoing, but there is evidence of platelet response after treatment with azathioprine, cyclophosphamide, cyclosporin A, danazol, dapsone, mycophenolate mofetil, vinblastine, vincristine, and the thrombopoietin receptor agonist (TPO-RA) drugs eltrombopag and romiplostim [6]. Side effects from these treatments were generally tolerable unless noted. However, many of these studies are old and have small sample sizes, so results should be repeated before any objective judgment of efficacy can be made.

Azathioprine

Azathioprine dosed at 150mg/day has been reported to be effective in chronic refractory ITP [24,25]. One retrospective study spanning a six-month period demonstrated a response rate of 71.4%, with complete response in 38% of patients [24]. A prospective study of 53 patients showed a 64% response, with 40% maintaining a response after one year [25]. A study in France reported a 40% response rate, 29% of those with a persistent response after discontinuing azathioprine [26]. Another retrospective study of 96 patients found that 54% of patients responded to azathioprine, though only 2% had a sustained response [11]. Leukopenia and increases in transaminases were reported, as well as alopecia, gastrointestinal effects, and increased risk of malignancy, specifically lymphoma [24,25]. However, the sample sizes for many of these studies were small, so further studies are needed to solidify the evidence for the efficacy and safety of azathioprine.

Cyclophosphamide

A prospective study of 20 patients treated with high-dose pulsed IV cyclophosphamide showed a 65% complete response. The most common side effect was neutropenia, but acute deep vein thromboses and a psoas abscess were also seen [27]. Another prospective study of 30 patients reported a complete response in 55% of patients with refractory ITP after splenectomy and in 50% of patients who had not received a splenectomy [28].

Cyclosporin A

In a retrospective study of 30 children under the age of 18, cyclosporin A was found to be effective, with a 57% complete response rate and a 23% sustained response after completion of therapy. Side effects included hirsutism and were typically tolerable and reversible [29].

Danazol

A prospective study of 47 patients with chronic ITP with refractory disease after splenectomy demonstrates a 22% response rate after treatment with danazol [26]. A smaller prospective study of nine patients shows an 11% response, with side effects including weight gain, arthralgias, headache, rash, amenorrhea, breast discomfort, and weakness affecting 67% of patients [30]. A third prospective study of 10 patients with refractory ITP reports only a transient rise in platelets in 10% of patients, with side effects in 60% of patients [31].

Dapsone

In a retrospective study of 38 patients (including 12 children), dapsone was found to have a 49% response rate and 41% complete response, though sustained response after six months was seen only in 5% (all adults). Side effects were seen in 13% of those patients, with 5% requiring discontinuation of the drug due to skin rashes [32]. There was a 55% response rate with a complete response in 38% of patients in another retrospective study of 42 patients. Side effects of skin rash, methemoglobinemia, sulfa allergy, and neuropathy were reported in 31% of patients, with 22% requiring discontinuation of the drug [33].

Mycophenolate Mofetil

A prospective study of 20 patients with refractory ITP showed an 80% response with a 45% complete response rate after therapy [34]. Another prospective study of 21 patients reported a 62% response rate with 24% complete response. In this study, 14% of patients reported mild nausea and diarrhea [35]. A retrospective study of 46 patients demonstrated a 52% response rate with 33% complete response. Nausea, vomiting, abdominal pain, and myalgias occurred in 8% of patients in this study [36].

Vinblastine

A prospective study of 13 patients reports a 13% response after continuous IV vinblastine [26]. Another prospective study of 43 patients with ITP showed 53% complete response to slow-infusion of IV vinblastine in patients with acute ITP, and 32% maintaining a response after one-two years. Of patients with chronic ITP, 17% had a complete response lasting at least six months [37]. A third prospective study of 16 patients demonstrated a 67% response and 17% complete response [38]. Adverse effects of vinblastine included leukopenia and peripheral neuropathy [37].

Vincristine

A prospective study of 24 patients with refractory ITP reported that 70% benefitted from a slow-infusion of vincristine [39]. A subsequent prospective study of 10 patients showed 72% response [40]. A more recent study of 10 patients with refractory ITP showed a 100% response with 60% complete remission after vincristine use for refractory thrombocytopenia due to ITP, autoimmune hemolytic anemia, and Evan’s syndrome [41]. A retrospective study of 62 patients described a 75% response after two months of vincristine therapy, with 51% maintaining the response after a year [42]. Another retrospective study reported an 86% initial response rate, with a sustained response after two years in 20% [43]. Peripheral neuropathy is one of the commonly reported side effects [20,43].

Thrombopoietin Receptor Agonists (TPO-RA)

One of the most recent clinical developments that has changed the landscape of chronic refractory ITP therapy is the introduction of thrombopoietin receptor agonists (TPO-RA). The two most widely used of these drugs are romiplostim, an Fc peptide fusion peptibody that stimulates megakaryopoiesis, and eltrombopag, a naphthalenesulfonic acid that also stimulates platelet production [44]. Both romiplostim and eltrombopag are US Food and Drug Administration (FDA) approved for adults with chronic ITP, and eltrombopag is approved for children with the same condition. Several studies have been conducted to explore this new treatment. A recent meta-analysis that studied eltrombopag for chronic ITP in adults and children showed that eltrombopag significantly improved platelet counts with a relative risk of 3.4 compared to untreated groups, and decreased the incidence of bleeding by a relative risk of 0.56. The analysis also demonstrated a decreased need for later rescue treatments for ITP [45]. A long-term, single-arm, open-label study of 292 adults with chronic ITP showed that romiplostim helped maintain a median platelet count of 50-200 x 10^9^/L and that a platelet response greater than 50 x 10^9^/L was achieved at least once by 95% of patients [46]. In a long-term study of 80 patients with chronic ITP treated with romiplostim, a platelet response of greater than 50 x 10^9^/L was seen initially in 74% of patients and was maintained two years later in 65% [47]. A retrospective study of 52 patients treated with romiplostim demonstrated a 78% response, though none had a sustained response [11]. In a prospective study of 70 patients, 93% had a peak platelet count greater than 50 x 10^9^/L after treatment with romiplostim, with remission in 32% after 24 weeks [48]. The side effect profile for these new treatments is still under question. Some reports cite minor side effects including headache, nasopharyngitis, upper respiratory infection, and fatigue, though more serious adverse events such as hepatotoxicity, arthralgias, vision changes, and severe fatigue have also been reported [49].

Ongoing studies including treatment with spleen tyrosine kinase inhibitors (Syk inhibitors) such as fostamatinib, anti-CD40 ligand, antihuman CD16 (FcγRIII) monoclonal antibody (GMA161), daclizumab, alemtuzumab, and avatrombopag are being conducted and will hopefully lead to treatments for chronic refractory ITP with a higher rate of complete and sustained remission and minimal side effects [5,44].

**Figure 1:**
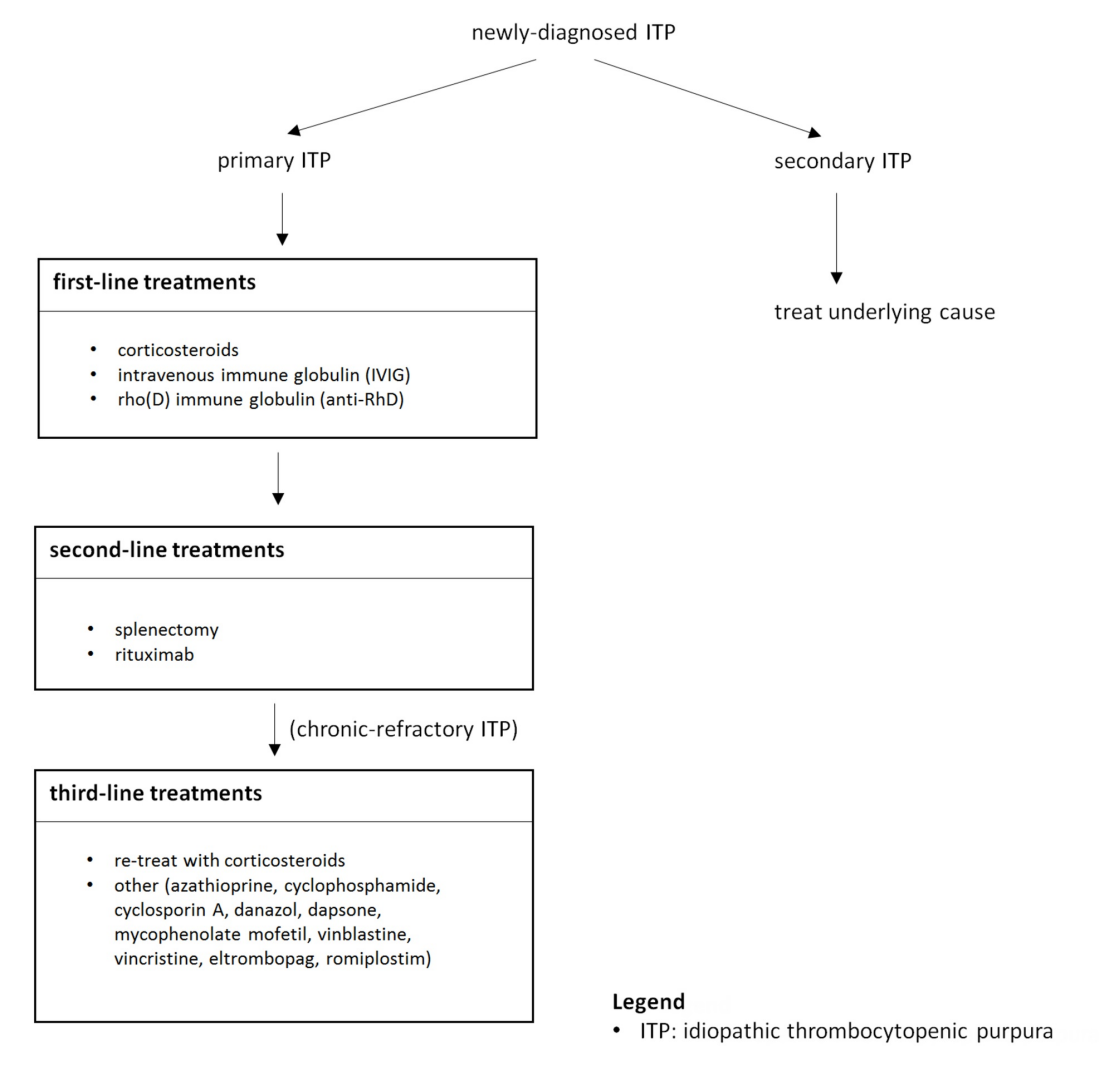
Stepwise treatment of ITP

A summary of the ITP treatment options is shown below (Figure 1).

## Conclusions

Treatment for ITP is complex and should be approached in a stepwise fashion. After a thorough literature search, we’ve found that the best first-line therapy for ITP remains corticosteroid use. In patients with steroid-refractory ITP, switching to or supplementing corticosteroids with IVIG or anti-RhD showed similarly significant response rates. Second-line therapies include splenectomy or the use of rituximab. While many patients obtain a response after the traditional first- and second-line therapies, chronic and refractory cases continue to drive the need for new and improved treatment options. Several treatment options have been described above, but there is not yet a universally accepted and effective third-line treatment in practice. In the meantime, patient- and institution-specific factors may drive the physician’s choice in third-line therapy.
